# Correlation of the presence and extent of loss of heterozygosity mutations with histological classifications of Barrett’s esophagus

**DOI:** 10.1186/1471-230X-12-181

**Published:** 2012-12-27

**Authors:** Eric Ellsworth, Sara A Jackson, Shyam J Thakkar, Dennis M Smith, Sydney Finkelstein

**Affiliations:** 1 RedPath Integrated Pathology, Inc, 2515 Liberty Ave, Pittsburgh, PA, 15222, USA; 2 West Penn Allegheny Health System, Division of Gastroenterology, Pittsburgh, PA, USA

**Keywords:** Barrett’s esophagus, Intestinal metaplasia, Indefinite for dysplasia, Low grade dysplasia, High grade dysplasia, Allelic imbalance, Loss of heterozygosity, Clonal expansion, Tumor suppressor gene mutation, Genomic instability

## Abstract

**Background:**

Recent advances in the management of Barrett’s Esophagus (BE) have placed greater emphasis on accurate diagnosis of BE as well as better prediction of risk for progression to esophageal adenocarcinoma (EAC). Histological evaluation of BE is particularly challenging with significant inter-observer variability. We explored the presence and extent of genomic instability in BE biopsy specimens as a means to add supplementary information to the histological classification and clinical decision-making related to early disease.

**Methods:**

We reviewed histology slides from 271 patients known to have BE. Using histological features as a guide, we microdissected target cell populations with various histological classifications of BE (intestinal metaplasia, “indefinite for dysplasia”, low grade dysplasia, or high grade dysplasia). DNA was extracted from microdissected targets and analyzed for loss of heterozygosity (LOH) using a panel of 16 LOH mutational markers associated with tumor suppressor genes at chromosomal loci 1p, 3p, 5q, 9p, 10q, 17p, 17q, 18q, 21q, 22q. The presence or absence of mutations and the clonality of each mutation were determined for each marker.

**Results:**

The presence and clonal expansion of LOH mutations was formulated into mutational load (ML) for each microdissected target analyzed. ML correlated with the histological classification of microdissected targets, with increasingly severe histology having higher ML. Three levels of mutation load (no ML, low ML, and high ML) were defined based on the population of microdissected targets histologically classified as intestinal metaplasia. All microdissected targets with dysplasia had mutations, with a high ML consistently present in high grade dysplasia targets. Microdissected targets histologically classified as intestinal metaplasia or “indefinite for dysplasia” spanned a range of no, low, and high ML.

**Conclusions:**

The results of this study reinforce the association of genomic instability with disease progression in BE. The presence and extent (clonality) of genomic instability, as assessed by mutational load, may assist histology in defining early stages of BE that are potentially at greater risk for disease progression. Assessment of mutational load using our panel of LOH mutational markers may be a useful adjunct to microscopic inspection of biopsy specimens, and thereby, improve patient management.

## Background

Barrett’s Esophagus (BE) is a pre-malignant condition that is associated with increased risk of esophageal adenocarcioma (EAC) [1]. EAC carries a relatively poor prognosis, with a 5-year survival rate below 13.6% [2]. In order to avoid progression to EAC, early intervention has become a primary focus. During BE carcinogenesis, genetic alterations that favor unregulated cell growth, such as activation of oncogenes and inactivation of tumor suppressor genes, cause morphologic changes in esophageal tissue [1]. Various studies have firmly established that mutational change involving tumor suppressor genes occurs at the histological onset of BE [3]. The more concerning histological categories of BE (i.e., the various grades of dysplasia) and EAC have been closely linked to progressive accumulation of multiple oncogene and tumor suppressor gene mutations [3].

Histological classification of BE is essential to disease management. Current guidelines define BE as specialized intestinal metaplasia with goblet cells [4]. These guidelines recognize the following histological classifications of BE in order of increasing severity: intestinal metaplasia, “indefinite for dysplasia”, low grade dysplasia (LGD), and high grade dysplasia (HGD). HGD is associated with greater risk of progression to EAC and, thus, warrants clinical intervention [5-8]. Recent guidelines call for LGD to be confirmed by a second pathologist prior to clinical intervention [4].

Although microscopic examination can readily identify BE and EAC, the various grades of dysplasia can be difficult to diagnose. The histological classification of dysplasia involves two especially challenging aspects: i) discriminating reactive epithelial atypia secondary to inflammation from the presence of true dysplasia; and, ii) distinguishing between the two different grades of dysplasia (LGD, HGD) [9-13]. Various guidelines for microscopic interpretation address these aspects, but, because of difficulties in defining precise thresholds of cumulative atypia, different pathologists may interpret the guidelines in different ways. In addition, a histological classification of “indefinite for dysplasia” is, at times, provided when cellular atypia is observed but does not clearly indicate dysplasia.

Consistently, many studies have shown inter-observer variability in the histological classification of Barrett’s dysplasia [9-13]. Such variability occurs among expert gastrointestinal (GI) pathologists, as well as those without GI specialization. In a recent study, up to 85% of patients who had LGD were re-diagnosed as having non-dysplastic BE or “indefinite for dysplasia” following pathology review [13]. The most pronounced variability in classification occurs around LGD, although variability can also be encountered in HGD. Because variability occurs even among expert pathologists, a substantial possibility for under- or over-diagnosis of dysplasia and its associated grades remains, thus complicating the decision of whether or not to perform treatment or alter the surveillance interval of the patient. The uncertainty in the diagnosis of histological dysplasia can limit stratification for the risk of EAC and make it difficult for a treating physician to know if a particular patient’s BE is at risk for progression.

A variety of endoscopic techniques exist for treating BE, including endoscopic mucosal resection, radiofrequency ablation (RFA), cryoablation, endoscopic submucosal dissection, and photodynamic therapy. Because these procedures are generally well tolerated, they are now routinely applied. Current American Gastroenterological Association (AGA) guidelines endorse endoscopic therapies in managing patients with high-grade dysplasia and “confirmed” LGD [1,4]; “confirmed” LGD is defined as diagnosis of histological LGD by at least two pathologists. Due to difficulties in histopathological diagnosis, debate continues regarding when endoscopic therapies should be performed in BE.

At present, there are no observable microscopic features of metaplasia that can determine if BE is likely to undergo disease progression to cancer or remain stable. The limited ability of histological features alone to identify those intestinal metaplasia cases likely to progress has led many to consider ablation and other interventions at the earliest stage of BE disease. This increased use of ablation has raised concerns over the associated healthcare economic burden [14,15]. Thus, supplementary diagnostic modalities that help to better characterize the early stages of disease would be a valuable addition to personalizing patient treatment and controlling these healthcare costs.

We aimed to better understand the relationship between histological changes in BE and the presence and extent of mutation acquisition. Previous work using microdissection-guided broad panel profiling for loss of heterozygosity (LOH) mutations in proximity to tumor suppressor genes has shown clinical utility for a variety of cancer applications [16-25]. We employed this method to characterize multiple histological sites within esophageal biopsy specimens from patients with BE. Areas of tissue with various histological classifications were tested for the presence and clonality of LOH across a panel of relevant genomic loci in order to characterize the overall LOH mutational load next to tumor suppressor genes. We hypothesized that increasing mutational load would correlate with increasingly severe histological classifications of BE. We show that an analytically objective and reproducible measure of the presence and extent of genomic instability, as assessed by mutational load, can assist histology in the characterization of BE.

## Methods

### Study cohort

We microscopically reviewed standard histological sections (4 μm thick) of archival, formalin-fixed, paraffin-embedded (FFPE) tissue from 271 patients histologically known to have BE. The study protocol was *Quorum Review* IRB approved (#26163) on June 8, 2011. Patients in the study cohort had previously undergone upper GI endoscopy and pathology review of biopsy specimens. We selected patients with a histological classification of intestinal metaplasia, “indefinite for dysplasia”, and various grades of dysplasia for inclusion in the study. Patients without evidence of BE were excluded, as were patients with intramucosal and/or invasive carcinoma.

### Microdissection

**Figure 1:**
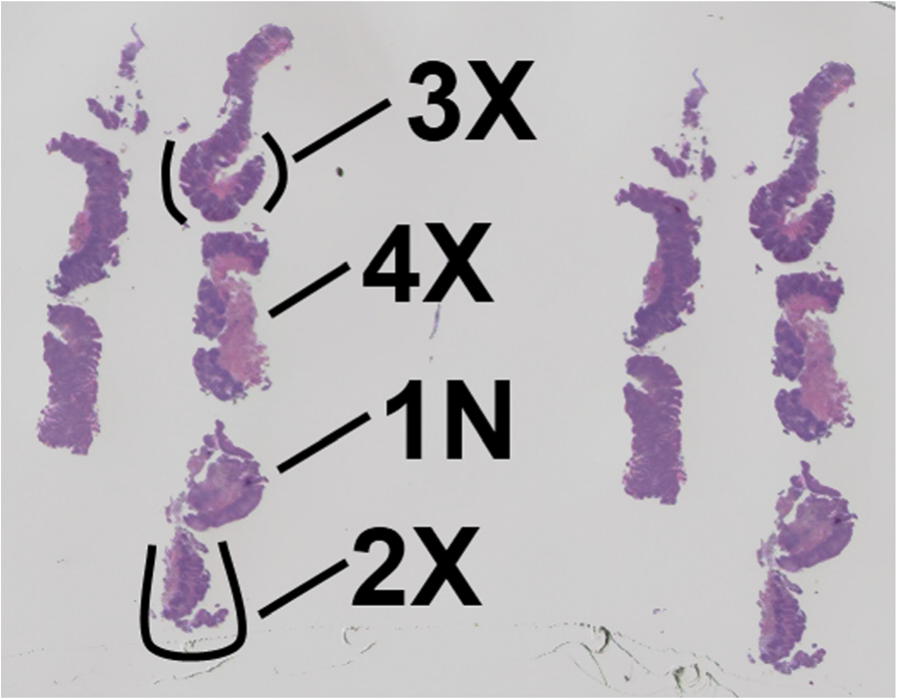
**Representative formalin-fixed, paraffin embedded (FFPE), hematoxylin and eosin (H&E) stained slide of Barrett’s epithelium.** Multiple re-cuts of several biopsies from the same patient are present on the slide. Multiple histological targets were microdissected from such slides for molecular analyses. 1N indicates a normal, non-Barrett’s epithelial microdissected target used as a baseline control. 2x, 3x, and 4x indicate individual microdissected targets containing Barrett’s epithelium.

Hematoxylin and eosin (H&E) stained, FFPE slides were used to guide microdissections of histologically classified targets from unstained, serial FFPE slides. Multiple distinct regions were microdissected from each slide to enrich for cells corresponding to distinct foci (targets) of histologically classified disease. Multiple microdissection targets were taken according to the availability of topographically separate tissue fragments, even when these fragments were from the same histological classification of BE (Figure 1). 1-3 targets of BE histology were microdissected from each patient’s FFPE biopsy specimen (2X-4X, Figure 1). Separate microdissected targets of histologically classified normal squamous epithelium (1N, Figure 1) and epithelium containing columnar cells that were not intestinalized (e.g., normal squamous and non-Barrett’s columnar mucosa) were examined as a baseline control for mutational markers. These targets were microdissected from the same FFPE slides as targets with various histological classifications of BE. Accuracy of microdissection was confirmed by microscopic review of post-microdissection stained slides.

### Histological classification

A histological classification for each microdissected target was assigned based on review of the specimens and accompanying histopathology reports from the original microscopic review. When consensus between pathologists was available, it was used as the histological classification for the microdissected target. When consensus was not available, we relied on the histological classification of the expert pathologist in our group. Microdissected targets were classified as non-Barrett’s epithelium and Barrett’s epithelium using the following histological classifications in order of increasing severity: normal squamous and columnar mucosa for non-Barrett’s epithelial microdissected targets, and intestinal metaplasia, “indefinite for dysplasia”, LGD, and HGD for Barrett’s epithelial microdissected targets.

### Detection of LOH

Using PCR and quantitative capillary electrophoresis, as previously described [19,20], we assessed 16 LOH markers associated with common tumor suppressor genes relevant to BE. The panel contained markers at the following 10 chromosomal loci (associated genes in parenthesis): 1p (*CMM1, L-myc*), 3p (*VHL, HoGG1*), 5q (*MCC, APC*), 9p (*CDKN2A*), 10q (*PTEN, MXI1*), 17p (*TP53*), 17q (*NME1*), 18q (*DCC*), 21q (*TFF1 and PSEN2*) and 22q (*NF2*) [19,20,26-28]. These markers have been analytically validated to detect LOH and were selected post-qualification studies using surgically resected EAC specimens microdissected at sites of intestinal metaplasia, dysplasia, and EAC, with histological classification at each site representing consensus of four GI pathologists. The results of such studies were used to define this smaller, more relevant panel of genomic loci, which includes LOH markers that had mutations in at least 20% of the surgical EAC specimens.

Before LOH was determined for each marker, DNA from all patients was examined to ensure it was adequate for PCR amplification using quantitative PCR (qPCR) results for housekeeping genes. This was performed on both lesional microdissection samples as well as non-neoplastic internal controls (normal appearing squamous and columnar mucosa and/or stromal tissue targets) all subject to equivalent formalin fixation and histological processing. PCR amplification and LOH analysis using quantitative capillary electrophoresis methods were then performed on all microdissection samples with adequate qPCR results.

To determine if each LOH analysis was assessable in each patient, the informativeness (heterozygosity) of each LOH marker in normal, non-Barretts tissue from each patient (1N, Figure 1) was evaluated to determine if the microsatellite repeats were homo- or heterozygous in length. In addition, preliminary studies characterized normal variability for each pairing of allele lengths examined for LOH in order to account for differing nucleic acid amplifications related to differences in allele length during PCR amplification. Normal squamous, non-Barrett’s epithelial microdissected targets were used to characterize this variability.

LOH was determined present when there was a degree of allelic imbalance that was equal to or beyond two standard deviations above the average difference in allele peak heights for DNA in non-neoplastic normal microdissection targets (1N, Figure 1). The degree (clonality) of LOH was quantitatively estimated using the ratio of allele peak heights, which is proportional to the amount of LOH mutated DNA (and cells) present in the sample [19,20]. Each microdissected target with LOH was tested in duplicate or triplicate to ensure reproducibility. LOH mutations were defined as high clonality when >75% of the DNA had LOH mutation and low clonality when 50-75% of the DNA had LOH mutation. When <50% of the DNA was mutated, no mutations were reported due to the analytical limit of detection of the assay, which was 50% for each LOH.

### Analysis and determination of mutational load

For each microdissected target, we determined the presence of each LOH mutation at a given genomic loci and whether or not each LOH mutation was low or high clonality (i.e. whether or not 50-75% of the DNA contained LOH or >75% of the DNA contained LOH). Proportional odds logistic regression (POLR) was used to assign a numerical value to low clonality mutations and to high clonality mutations. In performing POLR, various histological classifications of epithelia were grouped together (e.g. non-BE epithelium vs. intestinal metaplasia vs. LGD vs. HGD, including or excluding “indefinite for dysplasia” microdissected targets, or all non-dysplastic vs dysplastic, etc.) to determine the impact on the calculated values. Proportional values were also evaluated using FAL (fractional allelic loss), which is analysis for the proportions of low and high clonality mutations to the number of informative markers. All results from various analyses consistently determined a proportional value of 0.5 for low clonality mutations and 1 for high clonality mutations. These numerical values for low clonality and high clonality mutations were added together for all loci containing LOH in a microdissected target. The resulting cumulative value was defined as the mutation load (ML) for that microdissected target.

We examined the correlation between histological class and mutational load using Spearman rank correlation and calculated frequency distributions for each histological class within the study population. Levels of mutational load (ML) were established based on the frequency with which a particular level of ML was observed in microdissected targets histologically classified as intestinal metaplasia. The no ML level consisted only of intestinal metaplasia microdissected targets that lacked detectable mutations. The high ML level was defined as the level that captured 5% of intestinal metaplasia microdissected targets that had the highest level of ML. However, because ML was defined at discrete levels, only 4% of intestinal metaplasia microdissected targets were included by this cutoff. The low ML level included all intestinal metaplasia microdissected targets that had mutations but had an ML below the high ML cutoff. We used these levels of ML to evaluate the mutational load in other histological classifications. The frequency of mutations in various genomic loci of each microdissected target was also determined for each histological class.

## Results

### LOH mutational analysis

Esophageal biopsies were examined for LOH mutational profiles adjacent to tumor suppressor genes. Each FFPE biopsy slide was microdissected at multiple target sites as guided by histologically observed cellular morphology (Figure 1). Microdissections of distinct targets were performed on patient samples with various demographics (Table 1). There were 199 males and 72 females from which 568 distinct microdissection targets were analyzable.

**Table 1:** Demographics of patients included in study

**Age (years)**	**Male**	**Female**	**Total**
< 40	8	2	10
40-50	31	13	44
50-60	54	26	80
60-70	59	14	73
70-80	33	10	43
≥ 80	14	7	21
Total	199	72	271

The number of LOH mutations was determined in microdissected targets with various histological classifications. Table 2 summarizes the number of mutated LOH loci per microdissected target averaged for all targets across the range of histological classes examined. The number of mutated LOH loci increased with increasing severity of histological classification. Most LOH mutations were detected in HGD microdissected targets, and in those HGD targets a relatively high proportion of DNA (>75%) was found with these mutations (high clonality). While most mutations found in HGD targets were high clonality, mutations found in non-dysplastic histological classifications (intestinal metaplasia, “indefinite for dysplasia”) were typically low clonality. Compared to microdissected targets with Barrett’s histology, there were less mutations detected in targets histologically classified as normal squamous epithelium and epithelium containing columnar cells that were not intestinalized (columnar, non-Barrett’s epithelium), and importantly, there were no high clonality mutations found in these microdissected targets (Table 2).

**Table 2:** Summary of total mutations detected by each histological classification

**Histological classification**	**Total microdissected targets tested**	**Average number of mutated loci detected per microdissected target**	**Average number of low/ligh clonality mutations detected per microdissected target**		**Average mutational load**
			**Low**	**High**	
Normal Squamous	82	0.1	0.1	0.0	0.1
Columnar	77	0.6	0.6	0.0	0.3
Intestinal Metaplasia	216	1.5	1.2	0.3	0.9
Indefinite for dysplasia	138	2.0	1.7	0.3	1.1
Low grade dysplasia	39	3.5	2.7	0.8	2.2
High grade dysplasia	16	4.0	1.5	2.5	3.3

Mutations were observed across the entire panel of genomic loci examined. Table 3 summarizes the frequency of mutation in each genomic loci for microdissected targets within each histological class. Microdissected targets histologically classified with dysplasia had the highest frequency of mutations at 17p (*TP53*), with mutations present in 14/16 (88%) HGD targets, 27/39 (69%) LGD targets, and 49/138 (36%) “indefinite for dysplasia” targets. 9p (*CDKN2A*) was also more frequently mutated than other loci with 7/16 (44%) HGD targets, 20/39 (51%) LGD targets, and 45/138 (33%) “indefinite for dysplasia” targets displaying mutations. 

**Table 3:** Frequency of LOH mutations within microdissected targets by histological classifications

**Loci**	**Tumor suppressor genes**	**The number (percent) of microdissected targets with LOH mutations at each loci in each histological classification**					
		**Normal squamous**	**Columnar**	**Intestinal metaplasia**	**Indefinite for dysplasia**	**Low grade dysplasia**	**High grade dysplasia**
		**N = 82**	**N = 77**	**N =216**	**N = 138**	**N = 39**	**N = 16**
1p	*CMM1, LMYC*	1 (1%)	0	13 (6%)	20 (14%)	**11 (28%)**	**5 (31%)**
3p	*VHL, OGG1*	1 (1%)	4 (5%)	29 (13%)	24 (17%)	**20 (51%)**	**8 (50%)**
5q	*MCC, APC*	2 (2%)	8 (10%)	43 (20%)	25 (18%)	**11 (28%)**	**13 (81%)**
9p	*CDKN2A*	4 (5%)	4 (5%)	**56 (26%)**	**45 (33%)**	**20 (51%)**	**7 (44%)**
10q	*PTEN, MXI1*	0	11 (14%)	**54 (25%)**	**40 (29%)**	**12 (31%)**	2 (13%)
17p	*TP53*	1 (1%)	4 (5%)	35 (16%)	**49 (36%)**	**27 (69%)**	**14 (88%)**
17q	*NME1*	1 (1%)	7 (9%)	21 (10%)	33 (24%)	**17 (44%)**	**9 (56%)**
18q	*DCC*	1 (1%)	7 (9%)	41 (19%)	14 (10%)	**12 (31%)**	**4 (25%)**
21q	*TFF1, PSEN2*	0	3 (4%)	20 (9%)	18 (13%)	7 (18%)	0
22q	*NF2*	0	0	11 (5%)	3 (2%)	0	2 (13%)

Bold numbers indicate that at least 25% of microdissected targets tested with each histological classification had the indicated LOH mutations.

### Assessment of genomic instability

**Figure 2:**
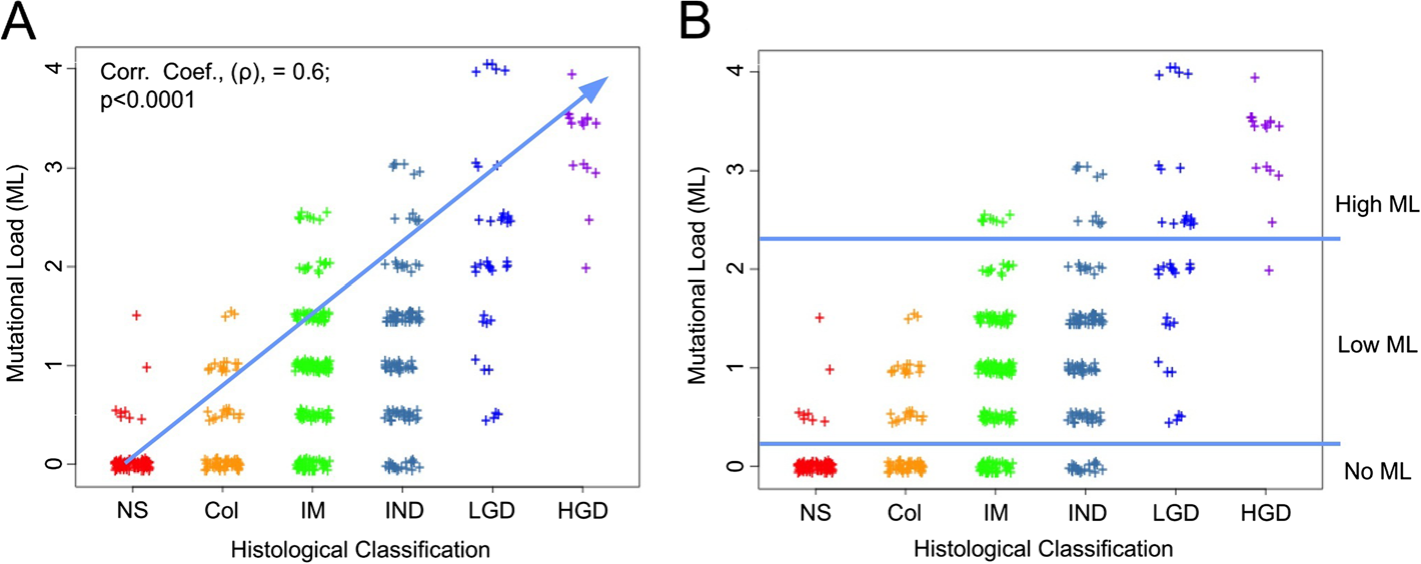
**Mutational load (ML) in microdissected targets by histological classification. A**.) There was a statistically significant correlation between increasingly severe histological classification and increasing mutational load (corr. coef., (ρ), =0.6; p<0.0001). **B**.) Levels of mutational load (no ML, low ML, high ML) were established within microdissected targets histologically diagnosed as intestinal metaplasia and then applied to other histological classifications. NS = normal squamous epithelium; Col = columnar, non-Barrett’s epithelium; IM = intestinal metaplasia; IND = “indefinite for dysplasia”; LGD = low grade dysplasia; HGD = high grade dysplasia; ML = mutational load.

Mutational load (Table 2, Figure 2) for a microdissected target represents the accumulation of clonally expanded LOH mutations next to tumor suppressor genes. High clonality LOH mutations were present when >75% of the DNA from the microdissected target had an LOH mutation, suggesting the presence of clonally expanded cells with that LOH mutation in the microdissected target. Low clonality LOH mutations were present when 50-75% of the DNA from the microdissected target had an LOH mutation. We used a semi-quantitative analysis that incorporated both the presence and clonality of all tested LOH mutations to provide an assessment of mutational load for a given microdissected target. In this system, we assigned low clonality mutations with a numerical value of 0.5 and high clonality mutations with a value of 1. These numerical values for low clonality and high clonality mutations were added together for all loci containing LOH in a microdissected target. The resulting cumulative value was defined as the mutation load (ML) for that microdissected target.

The mutational load for each microdissected target was correlated to the histological class of the target (Figure 2). Mutational load was positively correlated to histological classification, with the number and clonality of mutations increasing with increasingly severe histological classification (Figure 2A). Using the frequency with which mutational load was observed in intestinal metaplasia we established three levels of Mutational Load (ML) with respect to each histological classification (Figure 2B). The first level contained microdissected targets that lacked mutations and, as such, had no detectable ML. The second level contained microdissected targets with one low clonality mutation to two high clonality mutations with a mutational load greater than 0 but less than or equal to 2 and was defined as having low ML. The third level contained microdissected targets with greater than two high clonality mutations with a mutational load greater than 2 and was defined as having high ML.

Table 4 summarizes the proportion of microdissected targets for each level of mutational load in each histological class. The majority of histological microdissected targets with normal squamous epithelium and epithelium containing columnar cells that were not intestinalized (90% of normal squamous epithelial microdissected targets and 61% of columnar, non-Barrett’s epithelial targets) had no detectable ML (Table 4). Of the proportion of squamous and columnar microdissected targets that had mutations, all were low clonality mutations falling into levels of low ML (Table 2). 22% of histologically diagnosed intestinal metaplasia microdissected targets and 14% of “indefinite for dysplasia” microdissected targets had no ML, while the remaining proportion of intestinal metaplasia and “indefinite for dysplasia” microdissected targets had mutations with varying degrees of ML. All microdissected targets histologically classified as HGD and LGD had mutations with all targets falling into the low ML or high ML levels. All but one HGD microdissected target was characterized having high ML. Comparatively, only 4% of intestinal metaplasia and 9% of “indefinite for dysplasia” microdissected targets were characterized as having a high ML. 

**Table 4:** Frequencies of mutational load (ML) in microdissected targets by histological classification

**Histological classification of microdissected targets**	**Level of Mutational load (ML)**		
	**No ML**	**Low ML (1 low clonality – 2 high clonality)**	**High ML (> 2 high clonality)**
Normal squamous	74 (90%)	8 (10%)	0 (0%)
Columnar	47 (61%)	30 (39%)	0 (0%)
Intestinal metaplasia	48 (22%)	159 (74%)	9 (4%)
Indefinite for dysplasia	20 (14%)	106 (77%)	12 (9%)
Low grade dysplasia	0 (0%)	22 (56%)	17 (44%)
High grade dysplasia	0 (0%)	1 (6%)	15 (94%)

## Discussion

Accumulation of genomic instability within and next to oncogenes and tumor suppressor genes is associated with unregulated cell growth that can result in expansion of clonal cell populations and, ultimately, progression to cancer [3,29]. In this study, we surveyed the presence and extent of genomic instability by assessing the presence and clonality of LOH mutations adjacent to tumor suppressor genes in microdissected tissue targets with a range of histological classifications. The presence and extent of LOH mutations in tissue was correlated to its histological class. Microdissected targets, guided by morphological features, were taken at multiple sites, as available, in biopsy specimens. The overall number and extent of mutations were formulated into a mutational load that increased in correlation with increasingly severe histological classification. This correlation is consistent with the association of genomic instability with clonal expansion of cells and disease progression in BE.

BE microdissected targets with a histological classification of intestinal metaplasia were used to define three levels of Mutational Load (ML) with respect to each histological class: no ML, low ML and high ML (Figure 2B, Table 3). Levels of mutational load in tumor suppressor genes were established with respect to specimens histologically classified with intestinal metaplasia because i) the presence of intestinal metaplasia can be relatively reliably diagnosed [10,12]; and, ii) intestinal metaplasia is more prevalent in the clinical population than more severe histological classes of BE [30]. Therefore, defining levels of mutational load with respect to intestinal metaplasia makes the levels most relevant to the most frequent and reliable histology found in patients with BE. Microdissected targets with no ML were found in non-dysplastic histological classifications. Microdissected targets with low ML had relatively low levels of LOH mutational accumulation without evidence of clonal expansion of mutated cells. Microdissected targets with high ML had relatively high levels of LOH mutational load and clonal expansion of cells with these mutations. High ML was consistently found in higher levels of histological dysplasia; however, high ML was also seen in some cases with less severe histological classifications, such as intestinal metaplasia.

Specimens histologically classified as intestinal metaplasia and “indefinite for dysplasia” spanned a similar spectrum of LOH mutational load (no ML through high ML). We found that 78% of intestinal metaplasia microdissected targets had detectable mutations, despite the absence of morphological changes indicative of dysplasia. These results are consistent with a large body of work suggesting that DNA alterations in BE precede the overt morphological development of dysplasia [3,29]. Inflammatory responses can produce cellular changes that overlap those seen in true, confirmed histological dysplasia making differentiation of reactive atypia from true dysplasia difficult to determine. “Indefinite for dysplasia” microdissected targets tended to have higher mutation load than those with intestinal metaplasia, with mutations detected in 86% of microdissected targets (Table 4). Since these microdissected targets were histologically indefinite, some may, in fact, have more advanced histological disease than others. As with intestinal metaplasia, some “indefinite for dysplasia” microdissected targets may have mutations that precede morphological changes consistent with dysplasia. Therefore, mutational load analysis may provide additional information to aid in clinical diagnosis and management when such microscopic changes have yet to occur or are indefinite.

Lack of mutations (no ML) was observed in microdissected targets histologically classified as normal squamous epithelium and epithelium containing columnar cells that were not intestinalized (90% of normal squamous epithelial targets; 61% of columnar, non-Barrett’s epithelial targets) and in some of those classified with intestinal metaplasia (22%) and “indefinite for dysplasia” (14%) (Table 4). This lack of mutations (no ML) was not detected in any microdissected targets with histological dysplasia. In our previous experience across other organ groups using a similar LOH panel to examine mutations located near tumor suppressor genes, cell populations that lacked detectable LOH mutations were strongly correlated with benign, reactive processes [18,21,31,32]. Although gene panels employed in this study and previous ones are not a complete examination of the entire genome, the absence of clonally expanded LOH mutations next to the large number of tumor suppressor genes surveyed in our panel is strong evidence that the microdissected targets examined did not have extensive genomic instability. Therefore, microdissected targets that lack mutational load (no ML) are likely in the very early stages of neoplastic development or are morphologically displaying benign, reactive processes.

Microdissected targets that displayed low ML and were histologically classified as normal squamous epithelium and epithelium containing non-intestinalized columnar cells (10% of normal squamous epithelial targets; 39% of columnar, non-Barrett’s epithelial targets) could represent actual mutations within histologically normal appearing mucosa or detection of mutated DNA from adjacent cells or intercellular fluids. The squamous and columnar mucosal targets were microdissected from the same FFPE biopsy slides as those histologically diagnosed with BE, making it possible that mutations from the adjacent Barrett’s epithelium or intercellular fluids were detected. The mutational load in these squamous and columnar epithelial microdissected targets could also represent chromosomal aberrations that have yet to become morphologically visible by histology. The inability to assess the baseline mutational load in normal epithelium from a patient who lacks BE is a limitation of this type of study.

HGD is considered a severe premalignant event that requires clinical intervention, because it is associated with greater risk of progression to EAC [5-8]. In our study, applying cutoffs derived from intestinal metaplasia histological targets classified all but one HGD target as having high ML. This supports the association of high levels of genomic instability with more severe histological classifications of BE and is in line with the concept that patients with high ML may also be at greater risk of progression to EAC. Consistently, the presence of three or more DNA abnormalities in patients has been associated with a greater risk of progression towards cancer [33]. High ML may, therefore, provide support for associated interventions, even when histological classification of BE may be less than severe dysplasia (intestinal metaplasia, “indefinite for dysplasia”, LGD). High ML in less severe histological classifications of BE may be indicative of impending morphological changes that have yet to become histologically visible.

Our study is consistent with others that have described LOH mutations adjacent to *TP53* and *CDKN2A* tumor suppressor genes, which together have been associated with greater risk of BE progression to cancer [33-35]. When LOH mutations next to these genes have been analyzed in combination with additional DNA molecular markers for genomic instability, the risk of progression increases by over 4 fold at 10 years (relative risk of 38.7) [33]. Similar to these studies, we examined the mutational load in cell populations using a diverse DNA molecular panel to assess genomic instability. LOH adjacent to *TP53* and *CDKN2A* tumor suppressor genes were included in our panel and were found most frequently in microdissected targets with dysplasia (Table 3). Also included in our panel were 12 additional LOH markers next to other tumor suppressor genes relevant to BE and EAC [19,20,26-28]. Mutations were found in every one of the LOH markers in our panel (Table 3). Furthermore, the clonality of each LOH mutation was also assessed (Table 2). Increasing sizes of clones with genomic instability have been associated with increased risk of progression to EAC [35]. Consistently, the mutational load, which incorporates both the number and clonality of mutations, increased with increasingly severe histological class of BE, suggesting that mutational load is a relevant measure of genetic damage that can provide additional, objective information to the existing histological classification.

In addition to enhancing histological classification, this type of mutational profiling may also facilitate patient monitoring using sequential biopsies taken over varying periods of time prior to determining if ablation is needed. Furthermore, it can provide objective molecular information with respect to the success of ablation [36]. As with other forms of neoplasia, distinct clones of disease acquire distinct mutations, and a new clone is unlikely to have the same mutational profile as an existing clone. Incomplete elimination of the original clonal cell populations would be reflected in the same mutations persisting after ablation. In contrast, when the mutational profile in follow up biopsies differs from that of the initial biopsy, new clones of cells, as identified by different mutations, have likely evolved. When there is no evidence of mutations in follow up biopsies, complete eradication of atypical clones has likely been achieved.

A chief limitation in this study and all studies of BE is the variability associated with histological classification and the resulting lack of standardized histological classes for comparison to molecular results. Another limitation of this study concerns specimen type. Histology slides from biopsies are valuable specimens for studies such as this one, as they represent “real-world” specimens. However, biopsies are subject to sampling variation because, although current guidelines call for four-quadrant biopsies every 1cm across the region of dysplastic BE [4], in clinical practice, more limited sampling often occurs.

## Conclusions

The results of this study support the combined use of histological classification and mutational analysis to better evaluate BE. We demonstrate that various levels of mutational load (no ML, low ML, and high ML) adjacent to tumor suppressor genes exist within each histological classification (intestinal metaplasia, “indefinite for dysplasia”, LGD, HGD). HGD consistently has high ML, while other less severe histological classifications have a heterogeneous range of ML, spanning from no ML through high ML. According to the American Gastroenterological Association technical review of BE management, when initial biopsy specimens histologically show no dysplasia, indefinite dysplasia, or LGD, risk stratification for BE progression to EAC may be determined from collective clinical information, including the combined use of histological assessment and molecular biomarker information, if/when appropriate [1]. Assessment of biopsy specimens for levels of mutational load using our panel of molecular markers provides a relevant, objective, and reproducible measure of the presence and extent of mutational change that may provide an additional, quantitative dimension to histopathology for determining appropriate patient management.

## Abbreviations

EAC: Esophageal Adenocarcinoma; BE: Barrett’s Esophagus; LGD: High Grade Dysplasia; HGD: Low Grade Dysplasia; ML: Mutational Load; LOH: Loss of Heterozygosity; FFPE: Formalin Fixed, Paraffin Embedded; H&E: Hematoxylin and Eosin; GI: Gastrointestinal; RFA: Radiofrequency Ablation.

## Competing interests

RedPath Integrated Pathology (RedPath) sponsored this retrospective study. RedPath authors helped to design study and interpret/analyze data. Both RedPath authors and additional author (Shyam Thakkar, M.D.) participated in analyzing data, writing the manuscript, and reviewing the manuscript. RedPath also funded Rebbeca Palmer, Ph.D., who helped to prepare the article into the appropriate format according to guidelines for submission. Shyam Thakkar, M.D., received no financial support from RedPath. Sydney Finkelstein, M.D., is a founder of RedPath Integrated Pathology (RedPath). He is the current Chief Scientific Officer, is a shareholder, and is on the board of directors of RedPath. Dennis Smith, M.D., is the current Chief Executive Officer, a shareholder, and a member of the board of directors of RedPath. Eric Ellsworth, M.S., and Sara Jackson, Ph.D. are employees of RedPath. Shyam Thakkar, M.D., has no competing interests.

## Authors’ contributions

EE: Contributed to study design, data interpretation/analysis, statistical analysis, manuscript preparation /review, final approval of manuscript. SJ.: Contributed to data interpretation/analysis, manuscript preparation/review, final approval of manuscript. ST: Contributed to data interpretation/analysis, manuscript preparation/review, final approval of manuscript. DS: Contributed to data interpretation/analysis, manuscript preparation/review, final approval of manuscript. SF: Contributed to study design, data interpretation/analysis, manuscript preparation/review, final approval of manuscript.

## Pre-publication history

The pre-publication history for this paper can be accessed here:

http://www.biomedcentral.com/1471-230X/12/181/prepub
